# The William Goat

**Published:** 1880-12

**Authors:** 


### THE WILLIAM GOAT.

Mary had a William goat,
And he was black as jet;
He followed Mary 'round all day,
And liked her! you just bet!

He went with her to school one day,
The teacher kicked him out;
It made the children grin, you know,
To have the goat about.

But though old Whackem kicked him out,
Yet still he lingered near;
He waited just outside the door
Till Whackem did appear.

Then William ran to meet the man—
He ran his level best;
And met him just behind, you know,
Down just below the vest.

Old Whackem turned a somersault;
The goat stood on his head;
And Mary laughed herself so sick
She had to go to bed.

